# Supplementary material for: Identification of photoperiod-induced specific miRNAs in the adrenal glands of Sunite sheep (Ovis aries)
Source: Front Vet Sci. 2022 Jul 22;9:888207. doi: 10.3389/fvets.2022.888207 (PMC9354845; doi:10.3389/fvets.2022.888207)
Supplement: Supplementary file 1 [file Data_Sheet_1.ZIP › Supplementary file/miRDeep2 file.zip!/miRDeep2 file/Know_Structure/SP42A2/oar-mir-377.pdf]

miRBase precursor : oar-mir-377
Total read count : 27
oar-miR-377-5p read count : 16
oar-miR-377-3p read count : 1
remaining reads : 10

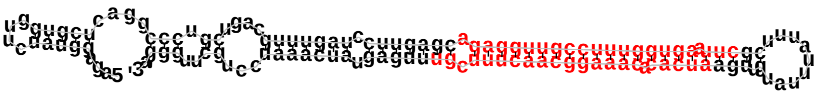

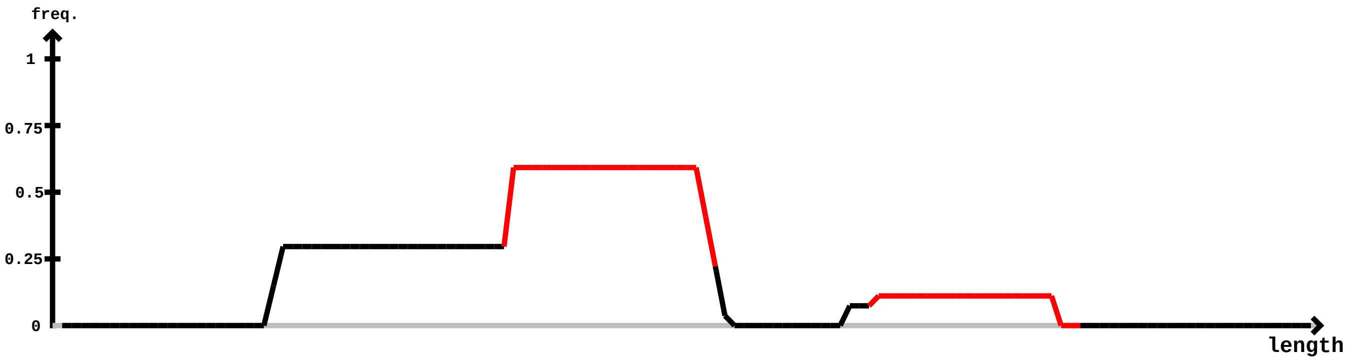


```
                                                                                                     oar-miR-377-3p
                                             oar-miR-377-5p
5'- agagguaucuuggugcucaggcccugcugacguuugauccuugagcagagguugccuuuggugaauucgcuuuauuuauguugaaucacacaaaggcaacuuucguuugaguaucaaauccugcuuggga   -3'    exp
    ...(((((....)))))....(((.((....(((((((.(((((((.(((((((((((((((((.(((((.........).)))))))).))))))))))))).))))))).)))))))...))..))).    reads     mm
    .....................cccugcugacguuugauccuugagc....................................................................................    4         0
    ......................ccugcugacguuugauccuugagc....................................................................................    4         0
    ..............................................agagguugccuuuggugaau................................................................    5         0
    ..............................................agagguugccuuuggugaauu...............................................................    5         0
    ..............................................agagguugccuuuggugaauuc..............................................................    5         0
    ..............................................agagguugccuuuggugaauucg.............................................................    1         0
    .................................................................................ugaaucacacaaaggcaacuuu...........................    2         0
    ....................................................................................aucacacaaaggcaacuuu...........................    1         0
```
